# Supplementary material for: Yogurt Alleviates Cyclophosphamide-Induced Immunosuppression in Mice through D-Lactate
Source: Nutrients. 2024 May 6;16(9):1395. doi: 10.3390/nu16091395 (PMC11085661; doi:10.3390/nu16091395)
Supplement: Supplementary file 1 [file nutrients-16-01395-s001.zip › nutrients-2881090-supplementary.pdf]

Supplementary Materials

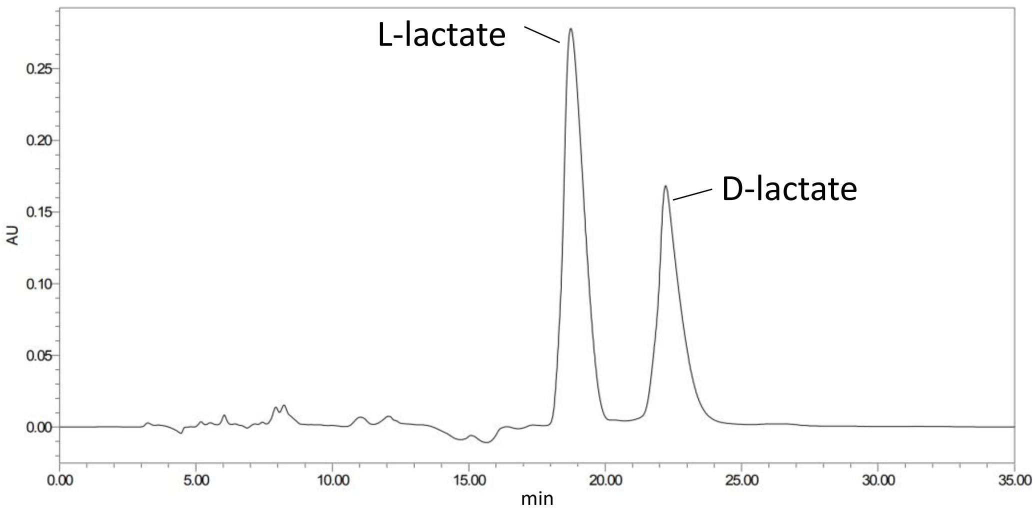

Figure S1. The chromatogram of lactate concentration in simulated commercial yogurt detected by high-performance liquid chromatography (HPLC).

Table S1. The lactate concentration in simulated commercial yogurt.

**Ingredients**	**Lactate Content (mg/mL)**	**Lactate Ratio (%)**
D-lactate	5.90±0.02	0.57±0.02
L-lactate	9.53±0.03	0.93±0.03
Total lactate	15.43±0.01	1.50±0.01

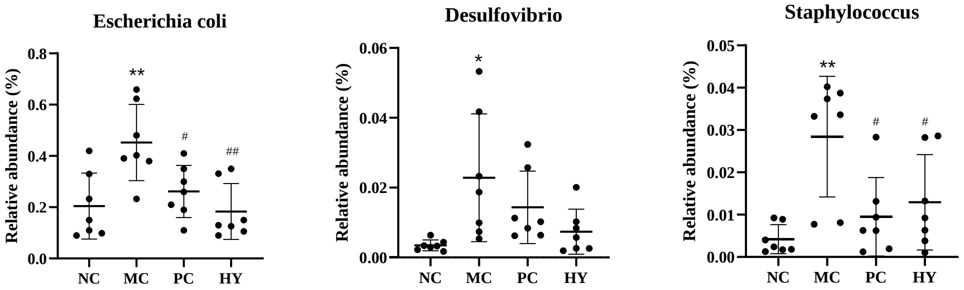

Figure S2. The relative abundance of Escherichia coli, Desulfovibrio and Staphylococcus in the fecal of CTX-induced mice. Statistical significance was evaluated by performing a one-way ANOVA followed by the Tukey test on the values presented as mean ± SEM. *$p < 0.05$, **$p < 0.01$, ***$p < 0.001$ vs. normal control group (NC). $^{\#}p < 0.05$, $^{\#\#}p < 0.01$, $^{\#\#\#}p < 0.001$ vs. model control group (MC).

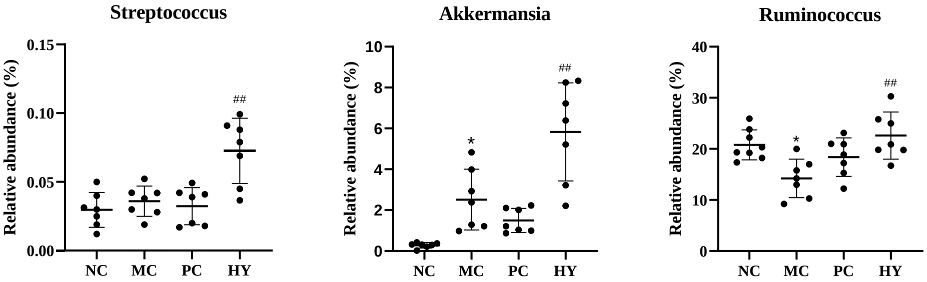

Figure S3. The relative abundance of Streptococcus, Akkermansia and Ruminococcus in the fecal of CTX-induced mice. Statistical significance was evaluated by performing a one-way ANOVA followed by the Tukey test on the values presented as mean ± SEM (n = 10). **p* < 0.05, ***p* < 0.01, ****p* < 0.001 vs. normal control group (NC). #*p* < 0.05, ##*p* < 0.01, ###*p* < 0.001 vs. model control group (MC).
